# Familial adenomatous polyposis: non-surgical management of large bowel disease: endoscopic and chemoprevention strategies

**DOI:** 10.1007/s10689-025-00480-w

**Published:** 2025-06-01

**Authors:** Maria Daca-Álvarez, Andrew Latchford, Maria Pellisé, Francesc Balaguer

**Affiliations:** 1 Department of Gastroenterology, Centro de Investigación Biomédica en Red de Enfermedades Hepáticas y Digestivas (CIBEREHD), Hospital Clinic de Barcelona, Institut d’Investigacions Biomèdiques August Pi i Sunyer (IDIBAPS), Barcelona, Spain; 2 Polyposis Registry, St Mark’s Hospital, Harrow, UK; 3 Department of Surgery and Cancer, Imperial College, London, UK; 4 Facultat de Medicina i Ciències de la Salud, Universitat de Barcelona (UB), Barcelona, Spain

**Keywords:** Familial adenomatous polyposis, Colorectal cancer, Endoscopic surveillance, Chemoprevention

## Abstract

Familial adenomatous polyposis (FAP) is a hereditary disorder caused by constitutional pathogenic variants in the *APC* gene, leading to the development of up to hundreds of colorectal adenomas and a near-inevitable risk of colorectal cancer (CRC) if untreated. Traditional management relies on prophylactic colectomy, but advances in endoscopic techniques and chemoprevention offer alternative strategies to delay or even avoid surgery. This review explores the role of endoscopic surveillance, polypectomy strategies, and chemopreventive agents in FAP management, evaluating their efficacy, limitations, and the need for personalized approaches.

## Introduction

Familial adenomatous polyposis (FAP) is a hereditary colorectal cancer syndrome caused by inactivating mutations in the Adenomatous Polyposis Coli (*APC*) gene. This genetic alteration leads to the development of hundreds to thousands of adenomas in the colon and rectum, with an inevitable progression to colorectal cancer (CRC) if left untreated [1–4]. With the advent of safe prophylactic colectomy, a significant improvement in life expectancy was observed [5] due to a reduction in mortality due to CRC. Surgery therefore became the standard intervention, effectively preventing CRC but often at the cost of altered bowel function and long-term complications [4, 6]. However, there has been major advancement in endoscopic techniques and medical prevention, which challenge the current management paradigm of the large bowel. [2, 6–7].

This review explores the latest non-surgical strategies for managing large bowel disease in FAP, with a focus on endoscopic interventions and chemoprevention, their effectiveness, limitations, and the emerging role of personalized surveillance protocols.

## Role of endoscopy in Fap management

Endoscopic management has emerged as a pivotal non-surgical strategy for controlling large bowel disease in patients with FAP. By facilitating the removal of adenomas and enabling close surveillance, endoscopic interventions not only provide essential monitoring for post-surgical patients but also serve as an effective means to minimize CRC risk in those seeking to delay or avoid colectomy [6, 8].Both the European Society of Gastrointestinal Endoscopy and the American Society for Gastrointestinal Endoscopy emphasize the critical role of endoscopy in managing FAP, particularly in the identification of high-risk lesions and the determination of optimal surveillance intervals [9, 10]. Endoscopic management is fundamental to the early detection and prevention of CRC, reinforcing its integral role in the multidisciplinary care of FAP patients [3]. 

### Endoscopic downstaging

**Fig. 1:**
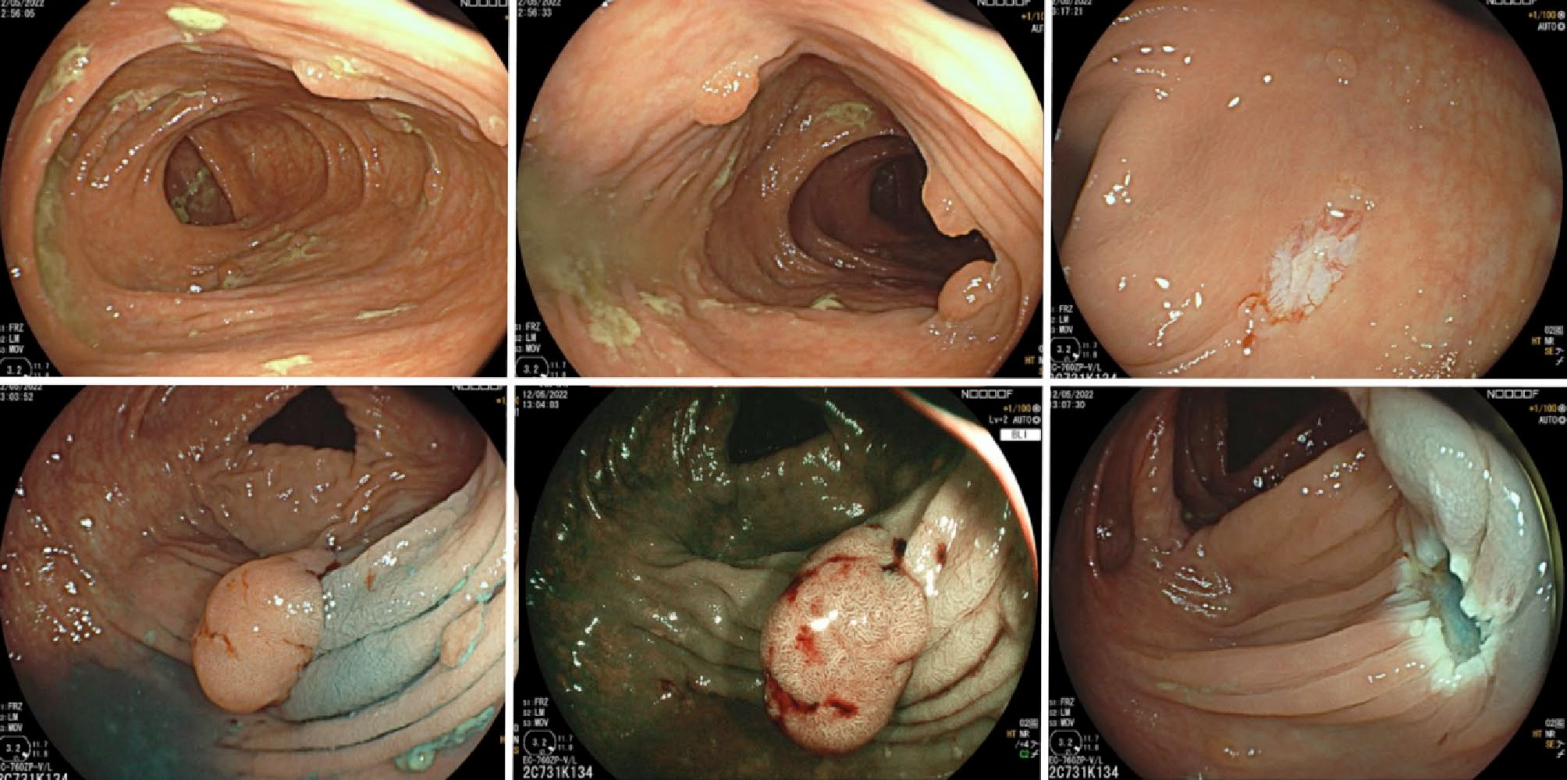
Patients with intact colon, endoscopic downstaging for delay or avoid surgery

The current standard of care for managing FAP in patients with an intact colon relies on prophylactic colectomy to prevent the progression to CRC [2, 6]. Surgical intervention has been proven to significantly reduce mortality and remains the definitive strategy for CRC prevention in these patients. However, in select cases, an intensive endoscopic approach may offer an alternative for patients with a mild polyp burden or those requiring delayed surgery due to clinical considerations. The J-FAPP Study III [6], a multicenter prospective trial conducted in Japan with 222 FAP patients undergoing an intensive downstaging polypectomy protocol showed that after five years, 90.4% of patients without colectomy retained their colon, suggesting the feasibility of this approach. However, the study included an heterogenous population with many patients > 50 years old that might not be representative of the FAP population. Moreover, the study involved a high procedural burden with 86.1% of patients undergoing > 5 colonoscopies in 5 years, with a mean number of polypectomies of > 500 per patient. Special concern about the study was that 21.1% of the population developed high-grade dysplasia or intramucosal carcinoma, which are high-risk CRC features in these patients, and 2 patients developed CRC during surveillance. Therefore, it is difficult to assess how feasible this approach would be in other healthcare systems and how acceptable it may be to patients; however, it highlights that endoscopic management in patients with an intact colon can serve as a short- to mid-term bridge before surgery, particularly in specific cases such as those with a high risk of desmoid tumors or in patients who decline surgery. In Fig. 1, an example of endoscopic downstaging can be observed. Another study has included endoscopic data specifically in those with a mild polyp burden, or phenotypic “attenuated FAP”. Of the cohort of 70, 40 patients had an intact colon. During colonoscopy surveillance no patients developed CRC, with a median age at last endoscopic procedure of 43 years (25 years − 73 years) [11].

### Surveillance after colectomy

Patients with FAP who undergo colectomy require lifelong endoscopic surveillance, as residual adenomas in the remaining bowel segments pose a risk for CRC. The choice between colectomy with ileorectal anastomosis (IRA) and proctocolectomy with ileal pouch-anal anastomosis (IPAA) is largely influenced by the severity of rectal polyposis at the time of surgery, and other factors such as the patient’s age, personal or family history of desmoid disease and sphincter function. A comparative summary of IRA and IPAA is provided in Table 1.

Patients undergoing IRA retain the rectum, which continues to be at risk for adenoma formation and malignant transformation. The reported cumulative risk of rectal cancer following IRA varies from 0.5 to 13%, with some patients eventually requiring secondary proctectomy due to uncontrollable rectal polyposis or cancer [12]. It is important to note that some of the studies mentioned on IRA include data from the pre-pouch period. At that time, patients with severe rectal polyposis underwent IRA, resulting in a higher risk of post-operative cancer. The establishment of pouch surgery and improvements in endoscopic surveillance may have contributed to a decrease in cancer risk over recent decades [13]. Indeed, in an international multicenter historical cohort study of FAP patients undergoing IRA or IPAA from 1990 to 2023, Bouchiba et al. [14] recently showed that among 366 patients with IRA, overall, only 8 patients (2.2%) developed rectal cancer after IRA. The estimated 10- and 20-year cancer incidence after IRA was 1.6% vs. 0.4%. In this population, reoperations, mainly for extensive polyposis, were performed in 39 (10.7%) patients with an IRA. Accordingly, the rectal remnant is a crucial site for surveillance, as adenomas can grow progressively over time, requiring frequent endoscopic removal. Surveillance strategies involve high-definition endoscopic assessments every 1–3 years, with the ultimate goal of preventing the need for secondary proctectomy by maintaining a manageable adenoma burden through timely endoscopic interventions [15–17] (Table 2).

**Table 1:** Comparison between ileorectal anastomosis (IRA) and ileal Pouch-Anal anastomosis (IPAA)

Characteristic	IRA	IPAA
Bowel segment	Rectum is retained	Rectum is removed; J-pouch constructed with distal ileum
Cuff	Not applicable	A rectal cuff (2–3 cm) is preserved near the anal sphincter
Functional outcomes	Better bowel function: fewer stools, better continence, less urgency	More frequent stools, higher risk of incontinence or urgency
Rectal cancer risk	Higher: due to residual rectal mucosa, requiring close surveillance	Lower: risk significantly reduced by rectal resection
Complications	Fewer complications	Risk of pouchitis and functional reservory issues
Fertility and sexual function	Preserved: pelvic nerves generally spared	May be reduced: pelvic dissection can affect fertility and sexual function

**Table 2:** Summary of surveillance recommendations from international guidelines

Author	Year	Patients with Ileorectalanastomosis (IRA)	Patients with ileal pouch-anal anastomosis (IPPA)
ESGE* Guideline (Van Leerdam ME et al.) [10]	2019	Every 1–2 years	Every 1–2 years
Yang J et al., 2020, ASGE* Guidelines (Yang J et al.) [9]	2020	6 months after surgery with 6 to 12 months further surveillance interval	12 months after surgery with 1–2 years further surveillance interval. 6 months if advance adenoma
British Guidelines (Monahan KJ et al.) [65]	2020	1–3 year	1–3 year

*ESGE: European Society of Gastrointestinal Endoscopy. *ASGE*: American Society of Gastrointestinal Endoscopy

**Fig. 2:**
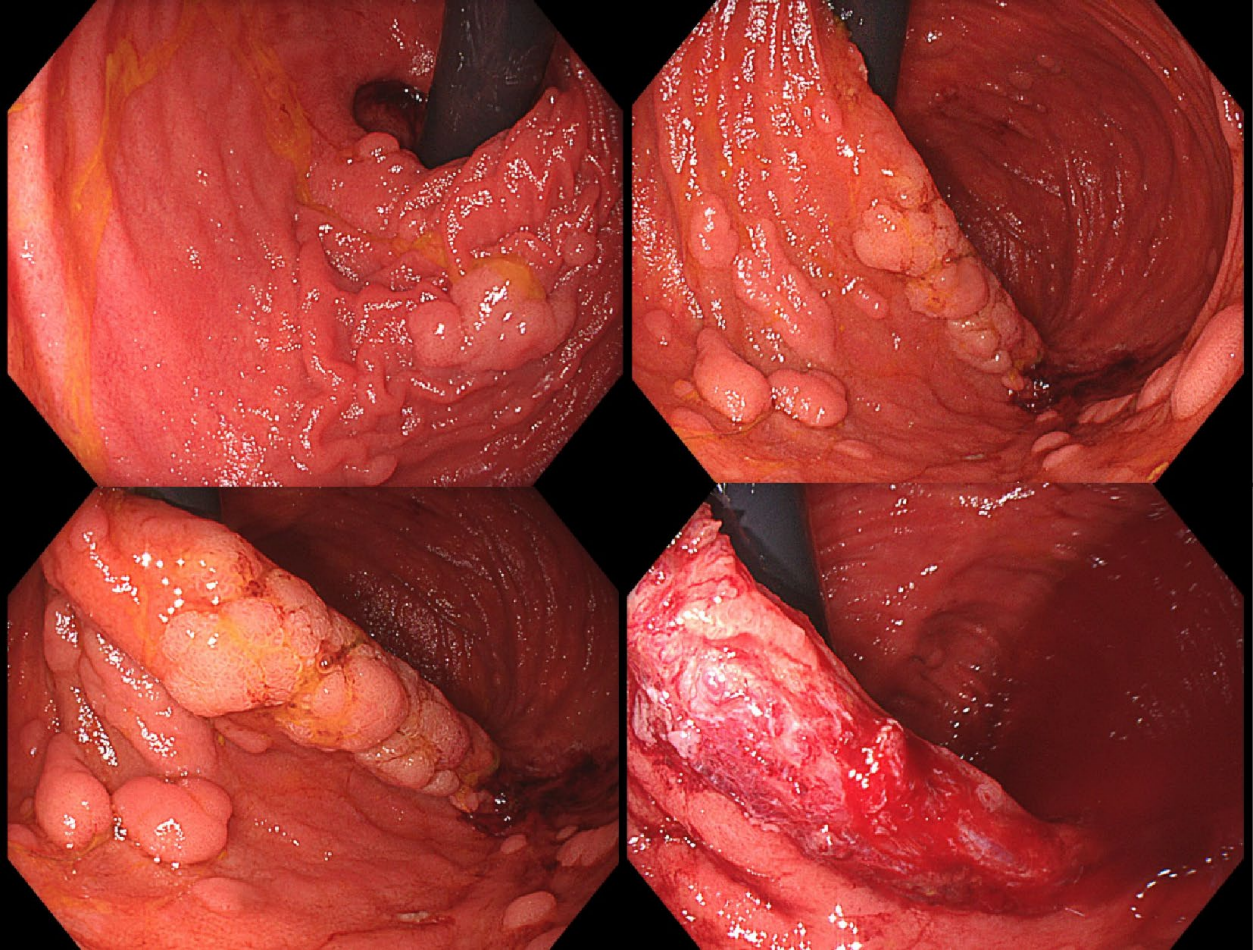
Patient with an IPAA under endoscopic surveillance, who underwent resection of a laterally spreading non-granular lesion (LST-NG) in the pouch

**Fig. 3:**
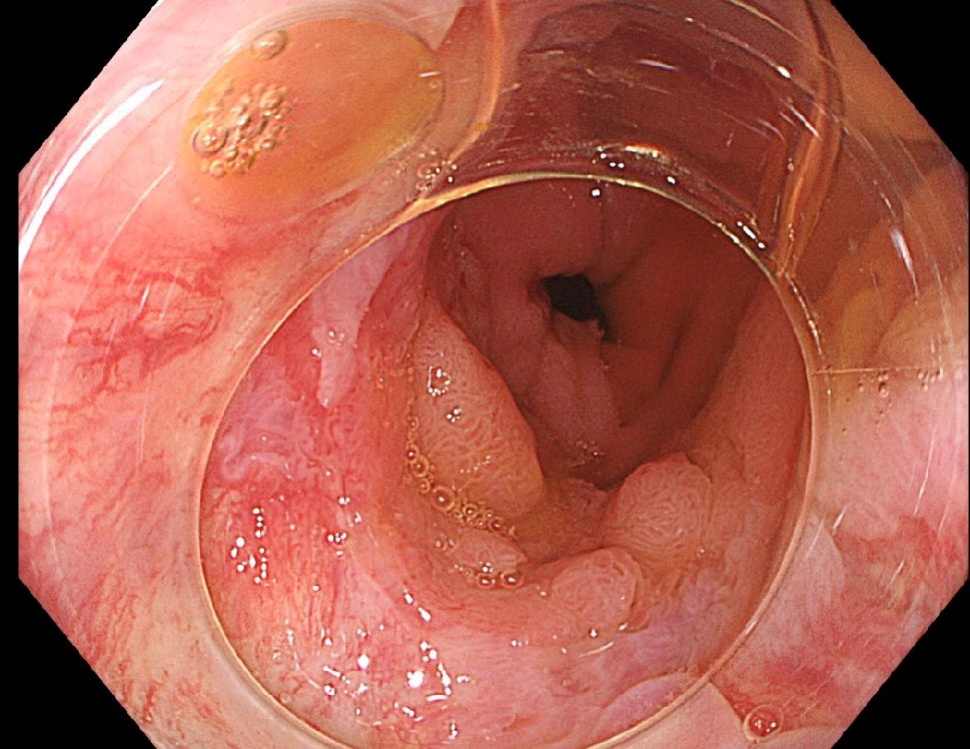
Patient with an IPAA under endoscopic surveillance, including a comprehensive exploration of the transition zone aided by a cap

For patients who undergo proctocolectomy with IPAA cancer risks range from 1.0 to 1.8%, and most cancers occur predominantly in the rectal cuff rather than pouch body. The rectal cuff, particularly in stapled anastomoses, is a site of increased risk due to the persistence of colorectal mucosa, which retains its adenoma-forming potential [18–21]. The study mentioned by Bouchiba et al. [14] in 319 patients with IPAA reported that only 0.9% of patients develop cancer in the rectal cuff/pouch, with an estimated 10- and 20-year cancer incidence after IPAA of 2.5% vs. 0.9%, respectively. Lifelong endoscopic surveillance is therefore required, with guidelines recommending endoscopic surveillance of the rectum or pouch with intervals ranging from 1 to 3 year (Table 1). Figures  2 and 3 illustrate an example of endoscopic surveillance in a patient with an IPAA (3).

**Fig. 4:**
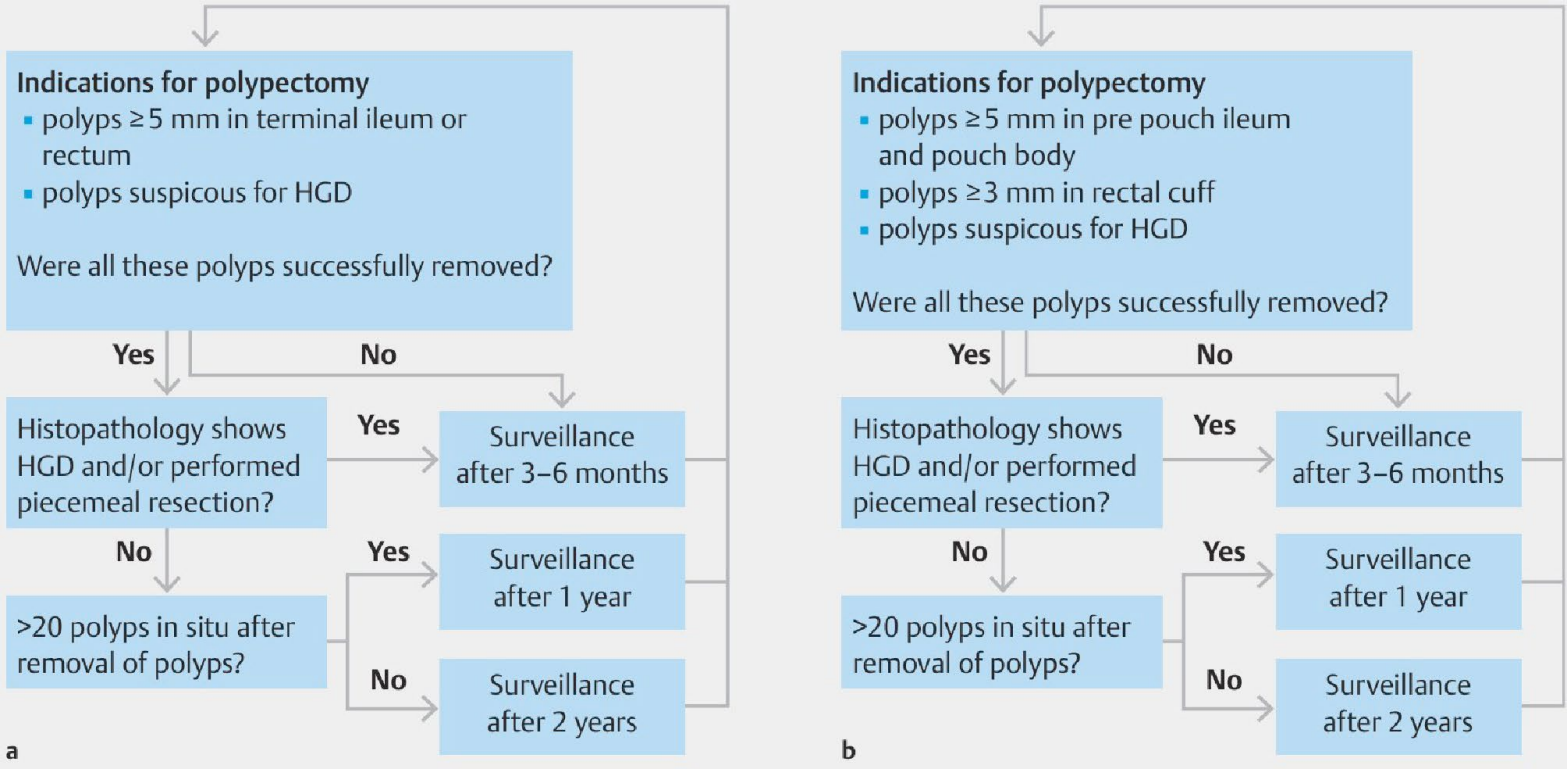
Personalized endoscopic surveillance strategies by the European FAP Consortium [8]. **A**) For IRA* **B**) For IPAA*. *IRA: Colectomy with ileorectal anastomosis. *IPAA: Proctocolectomy with ileal pouch-anal anastomosis

Given the variability in disease progression, the European FAP Consortium [8] has developed a consensus-based personalized endoscopic surveillance strategy, aiming to standardize surveillance intervals and polypectomy criteria to optimize patient outcomes. The proposed framework ensures that surveillance efforts are tailored to individual patient risk, ultimately improving long-term disease management in FAP patients. The proposed strategy for surveillance is detailed in Fig. 4 [8]. In summary, in patients with IRA surveillance intervals vary from 3 to 6 months to 2 years based on adenoma burden, dysplasia grade, and polypectomy completeness. Those with IPAA require monitoring for adenomas in the pouch and rectal cuff, with similar surveillance intervals tailored to disease severity. High-definition endoscopy with optional chromoendoscopy is recommended, and polypectomy is advised for adenomas ≥ 5 mm (≥ 3 mm in the rectal cuff). This protocol is undergoing a 5-year prospective evaluation across European expert centers to assess its effectiveness in reducing high-grade dysplasia, cancer incidence, and the need for proctectomy or pouch excision​.

## Chemoprevention

Chemoprevention involves using pharmacological agents to prevent, delay, or reduce disease progression, particularly in high-risk conditions such as FAP. Extensive research has evaluated a range of agents—from NSAIDs and selective COX-2 inhibitors to drugs targeting molecular pathways like mTOR—with the goal of developing accessible, cost-effective, and well-tolerated therapies that slow polyp progression and potentially delay or reduce the need for surgery. However, to date, no single agent or combination has been definitively shown to alter FAP’s natural course, with limitations arising from adverse effects, long-term safety issues, and inter-individual variability in response. In this review, we summarize current chemoprevention strategies, discuss the challenges in clinical trial design, and explore the evolving role of chemoprevention in the modern management of FAP.

### Anti-inflammatory drugs

Non-steroidal anti-inflammatory drugs (NSAIDs) like sulindac, celecoxib, rofecoxib and aspirin have shown effectiveness in promoting polyp regression in FAP patients.

*Sulindac* is a non-steroidal anti-inflammatory drug that inhibits COX-1 and COX-2. Daily doses of 150–300 mg have been shown to reduce the size and number of colorectal adenomas in FAP [22]. Prolonged therapy can preserve this benefit: a small prospective case series of 12 FAP patients reported no advanced adenomas after a median 64‑month follow‑up [23], whereas a larger observational trial of 54 patients (399 patient‑years; mean follow‑up 7.4 years) confirmed a rapid reduction in polyp burden in two‑thirds of patients [24]. Sulindac may delay colectomy in attenuated polyposis [25] and, as suppositories, a study with 15 post-colectomy patients showed complete adenoma reversion without relapse in 90% of patients after 33 months of follow-up [26]. However, stopping sulindac leads to rapid adenoma regrowth, indicating its effect is temporary [27] and rectal cancers have still been reported despite treatment [23, 27–29]. Moreover, a randomized, double-blind, placebo-controlled study of 41 young FAP patients showed no benefits in preventing colorectal adenomas formation [30]. Finally, sulindac sulfone (exisulind, a metabolite of sulindac without known effects on prostaglandin synthesis) show some efficacy in a phase I clinical trial but was limited by hepatotoxicity [31].

*Sulindac and erlotinib.* Studies have suggested that APC inactivation and epidermal growth factor receptor (EGFR) signaling promote COX-2 expression and the subsequent development of intestinal neoplasia. Erlotinib, an epidermal growth factor receptor (EGFR) inhibitor, works by blocking receptor signaling pathways that drive cell proliferation and survival. As a single agent, erlotinib has demonstrated promising chemopreventive effects in FAP. In a single-arm, multi-center Phase II trial, weekly administration of erlotinib (350 mg once per week) for 6 months led to a significant reduction in duodenal polyp burden (a mean decrease of 29.6%, 95% CI -39.6% to -19.7%, *p* < 0.0001) and modestly decreased lower gastrointestinal polyps [32]. Although 71.7% of participants experienced grade 2–3 adverse events, these events were generally low grade and well tolerated, suggesting that a weekly dosing regimen can strike a balance between efficacy and safety. Both sulindac and erlotinib possess chemopreventive activity; when administered together, their effects appear additive, achieving a larger reduction in adenoma burden than either agent alone. In a double-blind, randomized, placebo-controlled trial involving 92 FAP patients (22 with intact colon, 44 with ileal pouch anal anastomosis and 16 with ileo-rectal anastomosis), the daily combination of sulindac (300 mg/day) and erlotinib (75 mg/day) for 6 months resulted in a 70% reduction in colorectal polyp burden, with primarily mild skin-related side effects. This combination targets both the EGFR and COX-2 pathways, which are implicated in polyp development, thereby offering enhanced suppression of neoplastic progression in FAP [33, 34]. 

*Sulindac and eflornithine.* Molecular studies have suggested that inhibition of colorectal mucosal polyamines may be a promising approach to prevent CRC. Inhibition of ornithine decarboxylase (ODC) using low-dose eflornithine (also known as DFMO or CPP-1X) in combination with sulindac showed promising results [35]. A randomized trial in 171 patients assessed the efficacy of eflornithine (750 mg/day) and sulindac (150 mg/day), alone or combined for 48 months, in delaying disease progression in FAP. Disease progression, defined as a composite outcome of upper and lower gastrointestinal events, was not significantly reduced with combination therapy compared to monotherapy, and adverse events were similar across groups [36, 37]. However, a post hoc analysis found that the combination significantly delayed lower GI disease progression compared to monotherapy. Specifically, progression rates were 3.7% with the combination versus 17.0% and 19.6% with sulindac and eflornithine, respectively. When excluding the excision of large adenomas, the need for major surgery was nearly eliminated in the combination arm, highlighting its superior efficacy in delaying or preventing lower GI tract surgery [38].

*Celecoxib*, a selective COX-2 inhibitor, reduces gastrointestinal polyp formation by targeting the COX-2 pathway, which is upregulated in colonic adenomas and linked to malignant transformation. Preclinical models have shown that COX-2 inhibition decreases polyp number and size, while clinical trials in FAP patients demonstrated that high-dose celecoxib (400 mg twice daily) significantly lowers both polyp count and burden (by around 30%) and even slows duodenal polyposis [39–41]. A three‑year extension of the original trial subsequently reported zero colorectal cancers in 55 patients who continued celecoxib, versus two cancers in matched controls, although the study was not powered for this oncological end‑point [42]. Although long-term studies in pediatric FAP populations suggest a beneficial impact on delaying disease progression with an acceptable safety profile, concerns regarding cardiovascular toxicity with prolonged use have limited its broader adoption as an ideal chemopreventive agent. Similar results have been observed with rofecoxib, another COX-2 inhibitor, which also reduced polyp formation but raised concerns regarding increased thrombotic events with prolonged use [43, 44].

*Aspirin*, a non-selective COX inhibitor that irreversibly blocks both COX-1 and COX-2, has shown mixed results in FAP chemoprevention trials. Earlier studies using higher doses (e.g., 600 mg daily) in younger patients did not significantly reduce polyp numbers [45] though some reduction in polyp size was observed, whereas a double‑blind, randomized trial of low‑dose aspirin (100 mg/day) had to be stopped early after 3 of 17 treated patients developed serious gastrointestinal adverse events, including a giant anastomotic ulcer, aphthous colitis, and progressive anaemia [46]. More recently, a Japanese multicentre, randomized, controlled trial using low-dose aspirin (100 mg/day) in a two-by-two factorial design—alongside mesalazine—demonstrated that aspirin significantly suppressed the recurrence of colorectal polyps (≥ 5.0 mm), reducing the recurrence rate from 50 to 30% (adjusted odds ratio 0.37, 95% CI 0.16–0.86) over 8 months [47]. A commentary on this study was published underscoring that while the chosen endpoint (emergence of adenomas ≥ 5.0 mm after baseline clearance) was reasonable, trial design challenges remained, including ensuring even distribution of heterogeneous polyp burdens and achieving reproducible endoscopic measurements [48].Furthermore, the average patient age in that trial suggested many had attenuated FAP, in which progression over a short period may be minimal, potentially underestimating aspirin’s benefit. Despite these limitations and the modest effect observed, the widespread availability and low cost of aspirin make it a potentially attractive chemopreventive agent for FAP, meriting further long-term, well-powered studies with optimized endpoint measures.

Finally, *indomethacin* suppositories (50 mg once or twice daily) were evaluated in eight FAP patients who had undergone total colectomy with ileorectal anastomosis. After 4–8 weeks of treatment, six patients who initially presented with ≥ 10 rectal polyps showed a fall to < 5 polyps at the treated site, although the number of polyps increased again after therapy was discontinued. In four of these patients, rectal‑mucosa biopsies were obtained before and after treatment and epithelial proliferative activity was quantified by immunohistochemical staining for the Ki‑67 antigen (MIB‑1). The Ki‑67–labelling index rose significantly at the end of therapy compared with baseline, indicating increased mucosal cell proliferation despite the transient regression in polyp burden. These findings suggest that indomethacin suppositories can produce short‑term reductions in rectal adenomatosis in FAP, but the rebound in polyp number and the associated rise in proliferative activity highlight the need for longer‑term safety and efficacy studies [49].

## Phytoestrogens

Clinical studies in FAP have provided promising evidence for the chemopreventive role of phytoestrogens, especially when combined with insoluble fibers. Mechanistically, ER‑β is the predominant estrogen receptor isoform in normal colonic epithelium and functions as a tumour supressor; its expression declines early in the adenoma-carcinoma sequence, so restoring or activating ER‑β re‑establishes anti‑proliferative, pro‑apoptotic, and anti‑inflammatory signaling, such as down‑modulation of Wnt/β‑catenin and COX‑2 pathway, thereby counteracting early neoplastic growth. For example, a randomized, double-blind, placebo-controlled study by Principi et al. in patients with sporadic adenomas demonstrated that a mixture of phytoestrogens and fibers significantly increased ER-β protein levels and markers of apoptosis in colonic mucosa [50]. Similarly, Calabrese et al. administered a patented phytoestrogen-fiber formulation to FAP patients with ileal pouch-anal anastomosis for three months, resulting in marked reductions in the number and size of duodenal polyps alongside favorable modulation of gene expression, including up-regulation of ER-β and down-regulation of proliferative markers [51]. In a longer-term study, Tonelli et al. reported that treatment over 12 months not only sustained a decrease in colorectal polyp burden and dysplasia but also significantly elevated ER-β expression [52]. These clinical findings suggest that phytoestrogens, through selective ER-β activation, can effectively reduce polyp progression in FAP with minimal side effects, supporting their potential as a safe chemopreventive strategy.

### Fiber and vitamins

Vitamin C (ascorbic acid) has been investigated for its antioxidant and antineoplastic properties in FAP, but clinical trials have not provided convincing evidence of a chemopreventive benefit. In one study by Bussey et al., 3 g/day of ascorbic acid produced a temporary reduction in polyp area at 9 months in FAP patients with ileorectal anastomosis, yet this effect was not sustained at 12 months, and no significant change in polyp number was observed [53]. Similarly, DeCosse et al. examined a combination therapy including ascorbic acid (4 g/day), vitamin E, and varying doses of fiber over 48 months; while the high-fiber group showed lower polyp ratios at specific time points, the simultaneous alteration of multiple variables limited definitive conclusions about vitamin C’s role [54]. Additionally, preclinical data suggest that ascorbic acid’s efficacy might be contingent on the presence of *KRAS* mutations, implying that its potential benefit could be restricted to a subset of patients. To date, vitamin C has shown limited utility as a standalone chemopreventive agent in FAP, highlighting the ongoing need for novel strategies in this challenging field.

### Eicosapentaenoic acid (fish oil)

Fish oil, particularly in the form of eicosapentaenoic acid free fatty acid (EPA-FFA), has been investigated for its potential chemopreventive effects in FAP by downregulating mucosal arachidonic acid levels and reducing COX-2 expression. In a study by West et al., post-colectomy FAP patients with a rectal remnant showed a significant reduction in both polyp count (− 22.4%, *p* = 0.012) and polyp size (− 29.8%, *p* = 0.027) after 6 months of EPA-FFA treatment compared with placebo, with a tolerability profile similar to that of the placebo group [55]. While these results mirror the efficacy seen with COX-2 inhibitors, the exact cellular mechanisms remain to be fully elucidated [56].

The ongoing phase‑III EPA‑POL‑04 study [57] expands on West’s pilot in several key ways: it randomises 204 post‑colectomy FAP patients to 24 months of gastro‑resistant EPA‑FFA (2 g/day) or placebo and adopts clinically meaningful end‑points, the primary outcome is the cumulative number of rectal polypectomies (> 5 mm) over two years, while secondary outcomes include changes in InSiGHT polyposis stage, time to surgical intervention and long‑term safety/tolerability. By combining a much larger sample, a longer treatment window and surgery‑related end‑points, EPA‑POL‑04 will determine whether sustained EPA therapy not only shrinks polyps but also reduces the need for therapeutic polypectomy and slows clinical progression, thereby clarifying EPA’s role as a well‑tolerated, non‑toxic chemopreventive option in FAP.

### mTOR inhibitors

The mTOR pathway, a central regulator of cell growth, proliferation, and angiogenesis, has long been recognized as a promising target for chemoprevention in FAP. Preclinical studies have shown that mTOR inhibitors like rapamycin can reduce polyp proliferation in *APC* mutant models, and early clinical evidence, including a small case series, suggested beneficial effects on polyp size and dysplasia in FAP patients. A pilot study in four FAP patients with a retained rectum or ileal pouch demonstrated that six months of rapamycin treatment led to an 80% decrease in marked polyp size in most polyps and a reduction in overall polyp number, with notable induction of apoptosis or inhibition of proliferation in three out of four patients, albeit with several adverse events reported [58].

Recent evidence has focused on an enteric‑coated formulation, eRapa. In a Phase 2 open‑label study presented at the 2024 InSIGHT meeting, 30 adult FAP patients (with intact colons or post‑colectomy ileorectal anastomosis) were assigned to three dosing cohorts for 12 months [59]. Overall, 75% of patients were non‑progressors at 12 months, with a median 17% reduction in polyp burden. Efficacy differed by schedule: Cohort 1: 0.5 mg every other day: 70% non‑progressors; 15% median reduction; Cohort 2: 0.5 mg daily, one week on / one week off: 89% non‑progressors; 29% median reduction (best overall response); and Cohort 3: 0.5 mg daily, continuous: 67% non‑progressors; 10% median reduction. Treatment was generally well tolerated across all cohorts. Based on these results, a double‑blind Phase 3 trial will test the intermittent daily schedule (cohort 2) in a larger, high‑risk FAP population. Collectively, these data reinforce the promise of optimized rapamycin formulations to delay, or potentially avoid, the need for surgery in FAP.

### Other agents

- *Curcumin* has been evaluated for its chemopreventive effects in FAP with mixed results. In an early small open-label study, the combination of curcumin and quercetin was associated with a reduction in both the number and size of adenomas in patients with FAP. However, a subsequent randomized trial administering curcumin at 1500 mg twice daily failed to demonstrate a significant effect on intestinal adenomas. These contrasting outcomes suggest that while the combination with quercetin might enhance curcumin’s efficacy, curcumin alone at the tested dose does not provide a clear chemopreventive benefit in FAP [60, 61].
- *Black raspberries (BRBs*) are rich in chemopreventive compounds such as anthocyanins and ellagic acid. In FAP patients, a recent clinical trial using BRB suppositories—with or without additional oral BRB powder—demonstrated a significant reduction in rectal polyp burden and a modest decrease in polyp number. The treatment also decreased cellular proliferation and reduced promoter methylation of key tumor suppressor genes (e.g., p16) and miRNAs regulating the Wnt pathway. These findings suggest that locally delivered BRBs may offer a well-tolerated, non-toxic alternative for chemoprevention in FAP [62].
- *Imatinib*: Two published case reports describe patients with FAP who were being treated with imatinib for chronic myeloid leukaemia and, during therapy, showed a marked regression of their colorectal polyps [63]. Imatinib is a tyrosine‑kinase inhibitor that blocks BCR‑ABL, c‑KIT, PDGFR and, importantly for intestinal epithelium, EphB‑receptor signalling; by restraining these pathways it may curb intestinal cell proliferation at the earliest stages of tumor initiation. Although one case was confounded by previous treatments, the observed polyp regression points to a potential chemopreventive role for imatinib in FAP and justifies further systematic evaluation.
- *Venetoclax*: a case report was recently published of a 64-year-old man with FAP whose longstanding adenoma burden dramatically decreased after starting venetoclax—a BCL-2 inhibitor used for CLL. Over 13 years, he had numerous polyps resected, but following venetoclax initiation, his polyp count fell from 31 to just two small polyps. Given that BCL-2 is overexpressed in early colorectal tumorigenesis, these observations suggest that venetoclax may have a chemopreventive effect in FAP, warranting further investigation [64].

We summarize the current evidence from clinical trials of chemoprevention strategies in Table 3 (case reports and studies conducted solely in sporadic adenomas are not included).

**Table 3:** Summary table of clinical trials of chemopreventive agents in Familial adenomatous polyposis (FAP). (excludes case reports and sporadic‑adenoma studies)

Drug/Agent	Study Design and duration	Population	Primary endopoints	Key findings	References
ANTI-INFLAMMATORY DRUGS					
Sulindac	-Typical daily dose: 150–300 mg/day - Observational studies and small series (up to 64 months) - Larger trials (> 7 years’ follow-up) - One double blind RCT in young FAP (*n* = 41)	- FAP patients (intact colon) - Post-colectomy FAP - Attenuated FAP	- Reduction in number/size of colorectal adenomas - Delaying colectomy - Prevention of advanced adenomas	-Rapid and sustained adenoma regression in multiple studies (*p* < 0.05) - Effect lost after cessation - Rectal cancers still reported despite treatment - One RCT in young FAP: no benefit in preventing adenoma formation - Sulindac sulfone (exisulind) showed some efficacy but was limited by hepatotoxicity	[22–31]
Sulindac (Suppositories)	- Sulindac suppositories used in 15 post-colectomy patients - Follow-up: 33 months	- FAP patients with ileorectal anastomosis	- Complete adenoma reversion in retained rectum	− 90% had complete adenoma reversion - No relapse while on treatment, but rapid recurrence upon cessation	[26]
Sulindac + Erlotinib	1) Single-arm Phase II: erlotinib 350 mg/week × 6 months 2) Double-blind RCT (6 months): sulindac 300 mg/day + erlotinib 75 mg/day vs. placebo	- FAP patients with duodenal and/or colorectal polyps (intact colon/pouch/ileorectal anastomosis)	1) Reduction in duodenal polyp burden 2) Reduction in colorectal polyp burden	- Phase II: ~29.6% decrease in duodenal polyp burden (*p* < 0.0001), modest decrease in lower GI polyps - RCT: 70% reduction in colorectal polyp burden; primarily mild skin-related AEs	[32–34]
Sulindac + Eflornithine (DFMO)	- Randomized trial in 171 patients, 48-month follow-up - Daily doses: sulindac 150 mg + eflornithine 750 mg vs. monotherapies or placebo	-FAP patients (varying upper/lower GI involvement)	- Disease progression (upper and lower GI events) - Need for major surgery	- No significant difference on composite endpoint - **Post hoc**: combination significantly delayed lower GI progression (3.7% vs. 17–19.6% with monotherapies) and nearly eliminated need for major surgery	[35–38]
Celecoxib	- Typical high dose: 400 mg Twice Daily for 6 months - Also tested at 100 mg Twice Daily vs. 400 mg Twice Daily for 6 months in different FAP cohorts - Long‑term extension of original cohort: 400 mg BID, median follow‑up 3 years	- FAP patients (intact colon or post-colectomy)	- Reduction in colorectal polyp number, size, and burden - Delay of duodenal polyposis	- 28% decrease in mean polyp number with celecoxib (100 or 400 mg BID for 6 months) in 77 adult FAP patients (10) - 27% reduction in sum of polyp diameters with 400 mg BID in 112 adult FAP patients. -Extension: 0 CRCs on celecoxib vs. 2 CRCs in controls (under‑powered) - Protective effect enhanced in patients with polyp celecoxib levels correlating to serum levels. - Limitation: Cardiovascular toxicity concerns with prolonged use	[39–42]
Rofecoxib	- 25 mg/day for 9 months - Follow-up up to 16 months	- 21 colectomized FAP patients (rectal remnant)	- Reduction in number and size of rectal polyps - Prevention of advanced adenomas	- Efficacy comparable to celecoxib (*p* < 0.05), but withdrawn from market due to increased thrombotic risk	[42–44]
Aspirin	Various doses (100–600 mg/day) - Japanese multicenter RCT (100 mg/day, 8 months) with mesalazine	- FAP patients (younger cohorts in early studies; possibly attenuated FAP in the Japanese trial)	- Prevention of polyp formation/recurrence (≥ 5 mm)	- Earlier studies (600 mg): no significant reduction in polyp number, some size reduction - 100 mg trial halted for adverse events in one study - Japanese trial: reduced recurrence from 50–30% (OR 0.37, 95% CI 0.16–0.86)	[45–48]
Indomethacin (Suppositories)	- 50 mg once or twice daily - Small open-label (*n* = 8) for 4–8 weeks	FAP patients with total colectomy + ileorectal anastomosis	- Rectal polyp regression	- 6/8 patients reduced from ≥ 10 rectal polyps to < 5 - Polyps tended to recur after discontinuation	[49]
PHYTOESTROGENS (± INSOLUBLE FIBER)					
Phytoestrogens (± Insoluble Fiber)	- Patented phytoestrogen-fiber formula for FAP (IPAA), 3 months (51) - 12-month follow-up (52)	- FAP with ileal pouch-anal anastomosis (Calabrese, Tonelli)	- Changes in ER-β expression & apoptosis markers, polyp burden & dysplasia	- Significant increase in ER-β and apoptotic markers in colonic mucosa -Sustained decrease in colorectal polyp burden/dysplasia at 12 months; significantly elevated ER-β expression	[51, 52]
FIBER AND VITAMINS					
Vitamin C (± Fiber, Vitamin E)	- Bussey et al.: 3 g/day ascorbic acid, ~ 9–12 months(53) - DeCosse et al.: 4 g/day ascorbic acid + vitamin E ± varying fiber doses, 48 months(54)	- FAP patients with ileorectal anastomosis	- Change in polyp number/area	temporary reduction in polyp area at 9 months, not sustained at 12 months; no significant change in polyp number - Lower polyp ratios at certain time points in high-fiber arm, but multiple variables confounded conclusions about vitamin C’s effect	[53, 54]
EICOSAPENTAENOIC ACID (FISH OIL)					
**Fish Oil (EPA-FFA)**	- West et al.: 6-month, placebo-controlled study with ~ 2 g/day (dose details vary by formulation)	- Post-colectomy FAP patients with rectal stump	- Reduction in polyp count/size	-~22% decrease in polyp count (*p* = 0.012) and ~ 30% decrease in polyp size (*p* = 0.027) vs. placebo - Comparable tolerability to placebo - Mechanism linked to decreased COX-2 expression; larger trials (EPA-POL-04) underway	[55–57]
MTOR INHIBITORS					
mTOR Inhibitors (Rapamycin/eRapa)	1) Pilot study: 4 FAP patients, 6 months of sirolimus (rapamycin) (58) 2) Phase 2 open-label: 30 adult FAP patients for 12 months on eRapa (59)	-FAP patients with retained rectum or pouch (pilot) - Intact colon or post-colectomy with IRA (Phase 2)	Polyp size/burden - “Progression” (non-progression defined in Phase 2)	- ~80% decrease in marked polyp size, induction of apoptosis in 3/4 patients despite some AEs -75% were non-progressors at 12 months (median 17% reduction in polyp burden); best cohort achieved 29% median reduction. Generally well tolerated.	[58, 59]

### Challenges in the design of chemoprevention studies

Designing clinical trials for chemoprevention in FAP poses several challenges. First, the chosen outcomes must be clinically meaningful; it is insufficient to simply demonstrate a reduction in polyp burden unless this translates into tangible benefits such as delaying surgery, preventing advanced neoplasia, or reducing cancer incidence. To date, no randomized trial in FAP has been powered to use CRC incidence or mortality as a primary end‑point. Most studies (25 phase I–III trials since 1990) have instead relied on surrogate measures, such as polyp number, size or polyp burden, since CRC end‑point would demand thousands of patient‑years, long follow‑up, and high costs. Only three prospective studies [37, 42, 47] have reported cancer outcomes (celecoxib extension, sulindac + eflornithine registry follow‑up, and an observational aspirin cohort), but none was sufficiently powered or controlled to demonstrate a statistically significant risk reduction. Colorectal cancer could become a realistic end‑point in two scenarios: ultra‑high‑risk subgroups such as patients who decline colectomy, and registry‑based, protocolized “pragmatic” trials that link national FAP registries with electronic cancer records to capture long‑term events at lower cost.

Additionally, the heterogeneous nature of FAP patients, ranging from young, pre‑colectomy individuals to older post‑colectomy patients, complicates patient selection and endpoint interpretation. Furthermore, variability in endoscopic assessment, including inconsistent polyp counting and measurement, undermines the reliability of surrogate endpoints. Advances in imaging and central review protocols may help overcome these issues, but establishing robust, universally accepted endpoints remains a key challenge. Composite endpoints that bundle polyp burden with clinically decisive events (polypectomy load, escalation of surveillance frequency, progression in Spigelman/rectal stage, or need for surgery) could bridge the gap between short‑term mucosal change and long‑term cancer risk. Implementing such endpoints will require larger multicentre consortia, validated digital‑endoscopy metrics, and agents with enough expected effect size to justify prolonged follow‑up (Tables 3, 4).

**Table 4:** Challenges in chemopreventive studies design

Challenge	Explanation/Impact	Potential approaches
Clinically meaningful endpoints	Reduction in polyp burden must translate into delayed surgery, reduced neoplasia, or lower cancer incidence.	Focus on composite endpoints that combine quantitative polyp change with time‑to‑event measures (e.g., cumulative polypectomies, escalation of surveillance, need for surgery) and track long‑term clinical outcomes via registry linkage.
Patient heterogeneity	Wide variability in patient demographics (e.g., age, colectomy status) complicates patient selection and data interpretation.	Stratify patients based on disease stage and surgical history.
Variability in endoscopic assessments	Inconsistent polyp counting and measurement can lead to unreliable surrogate endpoints.	Implement standardized imaging protocols, AI-based strategies, and centralized reviews.
Lack of standardized endpoints	Without universally accepted endpoints, comparing study outcomes remains challenging.	Develop consensus guidelines for endpoint selection in FAP trials.
Long-term safety and tolerability	Extended treatment durations necessitate careful monitoring of adverse events and overall tolerability.	Incorporate robust safety monitoring and adaptive trial designs. Embed robust pharmacovigilance. Adaptive designs can help by dropping unsafe doses early, seamlessly expanding well‑tolerated regimens, and testing multiple agents under a single master protocol thereby limiting patient exposure to ineffective or toxic treatments.

### Which FAP patients might benefit from chemoprevention?

Currently, there is no formal guideline indication for chemoprevention in FAP, and treatment must be personalized. Indeed recent guidelines have concluded that there are insufficient data for chemoprevention drugs to be used in clinical practice, and their use should be in the framework of clinical trials [65]. Patients who might benefit most from chemoprevention are those who either decline further surgical interventions or in whom surgery is not ideal due to high morbidity risks. This includes individuals with extensive polyposis involving the rectum, ileal pouch, or duodenum—populations at continued risk for adenoma recurrence and progression despite previous colectomy. Additionally, younger patients with early-stage disease who are under regular endoscopic surveillance may also be candidates, as tailored chemopreventive strategies could potentially delay the need for surgery and reduce the frequency of invasive monitoring.

However, the feasibility of such a tailored approach is constrained by current practice guidelines. All major societies, NCCN (USA), EHTG / ESCP (Europe), NICE (UK) and JSCCR (Japan), still restrict chemopreventive drugs to research settings, and prophylactic colectomy plus scheduled endoscopy remain the standard of care. To move personalized chemoprevention into routine practice, several enabling steps would be required: (1) explicit, conditional recommendations in future guideline updates for clearly defined subgroups (e.g., attenuated FAP with low initial polyp load); (2) validated risk‑stratification tools that integrate age, *APC* genotype and baseline polyp burden to decide who should start therapy and when; (3) harmonized surveillance protocols, with digital or AI‑assisted polyp metrics, so that changes translate into consistent clinical decisions; (4) regulatory and reimbursement pathways (orphan‑drug status, pricing agreements) to secure access to promising agents such as high‑dose celecoxib, eRapa or EPA‑FFA. Complementary qualitative research is also needed to determine whether young, asymptomatic patients accept long‑term medication (and its monitoring requirements) in exchange for postponing surgery.

## Conclusion

FAP management is transitioning from a solely surgical approach toward integrating advanced endoscopic surveillance and chemoprevention. While endoscopy remains critical for early detection and removal of adenomas in both pre-colectomy and post-colectomy FAP patients, emerging chemopreventive agents—such as encapsulated rapamycin and sulindac combined with eflornithine—show promise in reducing adenoma burden and potentially delaying surgery. However, the true clinical benefit must extend beyond polyp reduction to preventing advanced neoplasia and CRC. Given the challenges of heterogeneous patient populations and variable endoscopic measurements, personalized treatment strategies and rigorous clinical trials are essential to identify the patients—particularly those who decline further surgery due to extensive polyposis—who are most likely to benefit from these emerging therapies.

## Data Availability

No datasets were generated or analysed during the current study.
